# Examining the role of brooding, distress, and negative urgency in dysregulated behaviors: A cross‐sectional study in treatment‐seeking young people

**DOI:** 10.1002/jclp.23366

**Published:** 2022-05-04

**Authors:** Dana Borg, Kate Hall, George J. Youssef, Elise Sloan, Liam Graeme, Richard Moulding

**Affiliations:** ^1^ School of Psychology Deakin University Geelong Victoria Australia; ^2^ Centre for Social and Early Emotional Development Deakin University Geelong Victoria Australia; ^3^ Center for Adolescent Health Murdoch Children's Research Institute Melbourne Victoria Australia

**Keywords:** behavioral dysregulation, emotion dysregulation, emotional cascade model, negative urgency, rumination

## Abstract

**Objective:**

Dysregulated behaviors including substance use, disordered eating, and nonsuicidal self‐injury (NSSI) have significant negative implications for individuals and health systems. It is therefore paramount to understand factors influencing behavioral dysregulation, to inform prevention and treatment approaches. The literature suggests that distress and rumination (brooding) prompt individuals to engage in behavioral dysregulation for distraction (Emotional Cascade Model), although these concepts have limited investigation in clinical, treatment‐seeking samples, particularly alongside negative urgency. This cross‐sectional study sought to examine the relationships of brooding, distress, and negative urgency with behavioral dysregulation, as well as the moderating effect of negative urgency between brooding and behavioral dysregulation, in treatment‐seeking young people.

**Method:**

A total of 385 treatment‐seeking young people completed cross‐sectional, self‐report measures of distress, rumination, negative urgency, and engagement in dysregulated behaviors (NSSI, alcohol use, drug use, binge eating, and purging) over the past 1−3 months.

**Results:**

Structural equation modeling revealed that only negative urgency, and not brooding or distress, had a significant positive relationship with behavioral dysregulation. Negative urgency did not significantly moderate the relationship between brooding and behavioral dysregulation.

**Conclusions:**

These findings reinforce the importance of considering negative urgency in the conceptualization, prevention, and treatment of behavioral dysregulation, and contribute to the knowledge of the relationship between brooding and various dysregulated behaviors within a treatment‐seeking sample.

## 1 INTRODUCTION

Dysregulated behaviors, such as nonsuicidal self‐injury (NSSI; e.g., deliberately injuring body tissue without suicidal ideation [Klonsky, 2007]); disordered eating (e.g., binge eating or purging resulting in distress and impairment [Buckholdt et al., 2014; Sloan et al., 2018]); and the binge use of drugs or alcohol, are a complex group of behaviors that are often comorbid (Blinder et al., 2006; Cucchi et al., 2016; Zlotnick et al., 1999), although what unifies this specific set of behaviors to often co‐occur requires further investigation. These behaviors are associated with a significantly heightened risk for suicide (Borges et al., 2017; Fliege et al., 2009; Franko & Keel, 2006), are difficult to control, result in functional impairment (Selby & Joiner, 2009), and have significant health and social implications, such as prolonged psychopathology, poorer physical health, and impaired occupational and interpersonal functioning (Mckellar et al., 2006; Perez & Warren, 2012; Turner et al., 2012). They also contribute to the significant expenditure of resources within healthcare systems (Hulse et al., 2001; Zanus et al., 2017). While a substantial literature purports the function of behavioral dysregulation is to regulate emotions by facilitating a shift in attention away from unpleasant emotional states (Brown et al., 2002; Hayes et al., 1996; Linehan, 1993; Nock & Prinstein, 2004), a unifying, multifaceted, theoretical model examining underlying cognitive, emotional, and trait‐like factors has yet to be used to collectively observe and understand these behaviors. Some models have proposed to explain behavioral dysregulation via impulsivity and behavioral expectancies (e.g., Acquired Preparedness model; Smith & Anderson, 2001), though they fail to take into account the often automatic, subconscious nature of behavioral dysregulation.

### 1.1 Emotional Cascade Model (ECM)

The ECM (Selby et al., 2008) theorizes the relationship between emotional and behavioral dysregulation occurs because of underlying cognitive processes, and that individual differences in the capacity to regulate emotions and subsequently behaviors are determined by these cognitive processes. The ECM suggests that “emotional cascades” are experienced by individuals enduring extreme surges of negative emotions, where people ruminate intensely about an emotion‐eliciting event (Selby et al., 2008). Rumination is a cognitive process characterized by the tendency to repetitively and passively focus on the causes, consequences, and symptoms of one's distress, without taking action to resolve or change the current problem (Nolen‐Hoeksema, 1991). An emotional cascade is presumed to result in a positive feedback loop of rumination and intense negative emotion, where both simultaneously intensify the other, creating an extremely aversive emotional state that individuals have difficulty tolerating (Kirkegaard Thomsen, 2006; Selby et al., 2008). The ECM proposes that individuals use dysregulated behaviors to shift their attentional awareness to physical sensations, thus distracting them from their ruminative thoughts (Selby & Joiner, 2009; Selby et al., 2008). Behaviors such as NSSI, binge eating, and substance use are believed to be commonly employed due to their effectiveness at short‐circuiting the self‐perpetuating emotional cascade, thus decreasing rumination and negative emotion, and providing individuals with immediate, emotional relief (Jungmann et al., 2016; Selby et al., 2008, 2009; Tuna & Bozo, 2014).

In spite of the novel contribution of the ECM to the theoretical understanding of behavioral dysregulation, a critical shortcoming of the existing literature is that it has not been widely tested in treatment‐seeking samples who exhibit clinically significant dysregulated behaviors. Given this model was developed to explain clinical phenomena, there are notable limitations in the current science underpinning the model, which is derived from nonclinical or community samples with low levels of behavioral dysregulation (Selby et al., 2008, 2009; Tuna & Bozo, 2014). This literature is arguably inadequate to determine whether the model holds clinical utility. Furthermore, the two preliminary studies in clinical samples focus predominantly on NSSI, which limits the conclusions that can be drawn about the applicability of the ECM to other forms of behavioral dysregulation (Selby et al., 2013, 2020). As such, research examining this model's clinical applicability in a clinical sample who engage in a variety of dysregulated behaviors is critical and importantly will inform whether rumination‐focused treatments are worthy of investigation for the treatment of dysregulated behaviors. The current study addresses the critical shortcomings of the existing literature on the ECM by examining several key components of this model within a clinical, treatment‐seeking sample who engage in various dysregulated behaviors concurrently.

While there has been a variety of methodologies employed to test the ECM, the majority have utilized cross‐sectional designs. Selby et al. (2008) first proposed and explored the observable and temporal relationships between emotional cascades and dysregulated behaviors including binge eating, reassurance seeking, and drinking alcohol to cope, using a two‐time point design with a sample of university students. Using structural equation modeling (SEM), emotional cascades (a latent construct comprising rumination, catastrophizing, and anger rumination) significantly predicted behavioral dysregulation, even when controlling for current psychological distress and deficits in adaptive emotion regulation (Selby et al., 2008). Using a subset of the initial sample who demonstrated heightened impulsive behaviors, relevant variables were readministered after 1 month. Even when controlling for changes in depression, anxiety, and emotion regulation, changes in rumination were related to changes in binge eating and reassurance seeking, but not drinking to cope. These findings support the temporal relationship between emotional cascades and dysregulated behaviors such as disordered eating and reassurance seeking (Selby et al., 2008).

Notably, each iteration of the ECM has measured emotional cascades differently; including additional cognitive processes alongside rumination or predominantly utilizing measures of general rumination and anger rumination. Consequently, definitional ambiguity surrounds an emotional cascade, and it is uncertain whether each construct independently significantly predicts behavioral dysregulation. Tuna and Bozo (2014) measured rumination, catastrophizing, and thought suppression as components of emotional cascades, predicting NSSI, binge eating, drinking to cope, and reassurance seeking in Turkish university students. This revised model was supported for females but not males. Gardner et al. (2014) examined NSSI and suicidal behaviors in a sample of incarcerated male offenders exhibiting traits of Borderline Personality Disorder (BPD), while broadening the measurement of emotional cascades to comprise a latent variable of rumination, that included brooding, anger rumination, catastrophizing, and rumination. Rumination significantly predicted self‐injurious behaviors, with rumination mediating the relationship between symptoms of BPD and behavioral dysregulation. Another extension of the ECM was applied to a German community sample demonstrating symptoms of obsessive‐compulsive disorder (OCD). Thought suppression, brooding, catastrophizing, and rumination, dysregulated behaviors, and compulsions, were included as components of the ECM, while also incorporating intrusions as a mediator between rumination and dysregulated behaviors (Jungmann et al., 2016). The model was supported in those with symptoms of both BPD and OCD, suggesting the ECM may be applied trans‐diagnostically. A more recent cross‐sectional study was the first to explore components of the ECM (depressive and anger rumination) in a clinical, treatment‐accessing sample of 91 participants with BPD who engaged in aggressive or NSSI behaviors (Martino et al., 2018). Findings demonstrated a positive relationship between both forms of rumination and these behaviors (Martino et al., 2018). Nonetheless, there remains a need for research examining various dysregulated behaviors within a clinical treatment‐seeking sample, to determine whether key components of the ECM, being rumination and distress, are robust enough to apply broadly to behavioral dysregulation. It is crucial to substantiate this relationship first, before adapting or broadening the scope of what an emotional cascade encompasses, as has been done in previous studies.

Additional studies have utilized ecological momentary assessments (EMA) and experimental designs to further understand the temporal relationships between psychological distress, rumination, and dysregulated behaviors. Initial findings from EMA studies suggested that when high levels of distress and rumination were recorded, individuals with heightened BPD symptoms had an elevated probability of engaging in dysregulated behaviors (Selby & Joiner, 2013). Furthermore, initial elevations in distress and rumination predicted subsequent increases, which exponentially predicted subsequent dysregulated behaviors (Hughes et al., 2019; Selby et al., 2016). These findings provide support for the reciprocal and progressive relationships between distress and rumination, which the ECM theorizes promotes dysregulated behaviors. Selby et al. (2013) similarly found via EMA that state rumination, particularly about the past, and distress, predicted daily NSSI. A recent EMA study found that the ECM accurately predicted BPD symptomology and behavioral dysregulation in a clinical sample (Selby et al., 2020). However, this sample was small and inclusive criteria related to current engagement in NSSI only, which may have excluded those who do engage in other forms of behavioral dysregulation. In the few experimental studies testing emotional cascades, findings have been mixed. Rumination inductions have demonstrated an increase in distress in those with a history of NSSI or disordered eating (Selby et al., 2009), although these increases were not always significantly different from controls (Arbuthnott et al., 2015).

While the methodology used to explore emotional cascades has progressed, several fundamental questions remain. First, the ECM has been examined in very few clinical samples, and the literature has predominantly focused on NSSI. Without testing key components of the ECM in a large clinical treatment‐seeking sample, where these symptoms are frequently observed, it cannot be assumed that the ECM is valid in explaining clinical phenomena, such as various forms of behavioral dysregulation (Chapman et al., 2005; Heatherton & Baumeister, 1991; Linehan, 1993). Second, inconsistent approaches to measuring an emotional cascade, and varied conceptualizations of rumination, have been included in studies (Gardner et al., 2014; Jungmann et al., 2016; Selby et al., 2008, 2009; Tuna & Bozo, 2014). Further limitations to the measurement of constructs in the literature include grouping varied conceptualizations of rumination together (e.g., depressive rumination, anger rumination), and incorporating additional constructs into the ECM (e.g., catastrophizing, suppression). Consequently, the existing literature has failed to ascertain the role of each construct independently, and there is definitional ambiguity surrounding what in fact encompasses an emotional cascade. To clarify the role of depressive rumination, especially in dysregulated behaviors so that the theoretical understanding of the ECM is enhanced, and to address the limitations of Selby et al.'s (2016) study, the present study intended to focus on one concentrated conceptualization of rumination, rather than grouping various measurements together.

### 1.2 Rumination: Brooding and reflection

The definition proposed by Nolen‐Hoeksema (1991) encompasses two distinct dimensions of rumination: brooding, which involves a passive focus on symptoms and a comparison of one's current situation with an unachieved standard; and reflection, which refers to intentional thoughts focusing on problem‐solving to relieve depressive symptoms (Treynor et al., 2003). Studies have consistently demonstrated that engagement in brooding is a risk factor for depression and suicidality (Crane et al., 2007; Mezulis et al., 2011), while reflection may actually be a protective factor against these outcomes (Burwell & Shirk, 2006). Given their well‐demonstrated effect on mood and suicidality, research into these dimensions has examined their relationships with varying dysregulated behaviors, though findings have been inconsistent. This is particularly apparent when examining the relationship between substance use and rumination. Willem et al. (2011) found that adolescents who engaged more in brooding and less in reflection were more likely to use substances, though longitudinal analyses contradicted these findings (Willem et al., 2014). Additional longitudinal studies have also failed to find a significant relationship between either rumination subtypes and overall substance use, though there has been some support for the relationship between brooding and problematic marijuana use (Adrian et al., 2014). Knowledge about the relationship between rumination subtypes and NSSI is even more scarce and uncertain, with findings indicating that despite engaging in high levels of both subtypes, a significant association was found only between those with a history of NSSI and reflection, and not brooding (Polanco‐Roman et al., 2015). Many studies have failed to distinguish between subdimensions of rumination when evaluating their role in binge eating (Breithaupt et al., 2016; Naumann et al., 2015). However, one study which did distinguish these forms found that brooding, but not reflection, interacted with body dissatisfaction to significantly predict binge eating (Gordon et al., 2012). No studies appear to have examined the subtypes of rumination and their role in predicting purging. Given these mixed and limited findings, the present study endeavors to clarify the role of brooding in a range of dysregulated behaviors in a clinical sample of treatment‐seeking young adults. Clarifying the role of brooding in dysregulated behaviors will further the theoretical understanding of what encompasses an emotional cascade, by determining whether brooding is a relevant component, or not. The present study simultaneously considers negative urgency, an underlying trait‐like factor, previously implicated in dysregulated behaviors.

### 1.3 Negative urgency and behavioral dysregulation

Negative urgency, which is the disposition to act rashly in response to negative affect (Lynam et al., 2007), is one of five subdimensions measured within the Negative Urgency, (lack of) Premeditation, (lack of) Perseverance, Sensation‐seeking, and Positive Urgency (UPPS‐P) Impulsive Behavior Scale (UPPS‐P; Lynam et al., 2007). Negative urgency has been demonstrated to be the subdimension most strongly implicated in impulsive behaviors, accounting for the broadest range of psychopathological symptoms often characterized as impulsive (e.g., BPD traits, disordered eating, substance use, and suicidality/NSSI) (Berg et al., 2015), and has been implicated in poorer treatment outcomes for substance use disorders (Hershberger et al., 2017). Given the established role of negative urgency in behavioral dysregulation, it is paramount to account for negative urgency alongside key components of the ECM, otherwise the role of rumination more broadly in the ECM is at risk of being overstated. Theoretically, negative reinforcement is assumed to drive the association between negative urgency and dysregulated behaviors. Individuals with high negative urgency who engage in behaviors such as substance use, disordered eating, and NSSI are presumably acting on a strong and immediate need to avoid aversive emotional stimuli (Berg et al., 2015). Additionally, Dick et al. (2010) propose that behaviors reinforced by negative urgency also occur due to depleted cognitive resources, whereby the experience of strong negative affect reduces ones' capacity to make effective choices or cope adaptively with difficult emotions. These explanations have marked similarities to Selby et al.'s (2008) ECM, whereby dysregulated behaviors are driven by a strong need to avoid aversive cognitive and subsequently emotional stimuli.

Given previous research finding a significant relationship between rumination and negative urgency (Valderrama et al., 2016), these commonalities could indicate that these processes overlap and contribute to deficits in emotion regulation, with negative urgency also contributing to dysregulated behaviors within the ECM. Due to the dearth of studies examining the constructs of negative urgency and integral components of the ECM such as rumination simultaneously, exploration of negative urgency alongside rumination has been called for to understand the unique role urgency has in predicting dysregulated behaviors (Berg et al., 2015; Wang & Borders, 2018). Interestingly, the first application of the ECM by Selby et al. (2008) included urgency as an indicator of the behavioral dysregulation latent variable, rather than including this in the Emotional Cascades latent variable as a predictor. Including urgency as a behavioral outcome variable alongside actual dysregulated behaviors conflicts with the UPPS‐P model's definition of urgency, in which it is conceptualized as a personality trait that predicts impulsive responses. All subsequent studies on the ECM have omitted negative urgency (Gardner et al., 2014; Jungmann et al., 2016; Selby et al., 2009; Tuna & Bozo, 2014). There have been only two studies that have explored the indirect effects of brooding and psychopathology symptoms, such as suicide risk and eating disorder symptoms through negative urgency (Valderrama et al., 2016; Wang & Borders, 2018). The first found no evidence of negative urgency mediating the relationship between brooding and suicide risk (Valderrama et al., 2016). The latter found negative urgency mediated the cross‐sectional, but not the longitudinal relationship between brooding and disordered eating in the nonclinical participants, while there was no evidence of mediation in the clinical sample cross‐sectionally or longitudinally (Wang & Borders, 2018). While mediation analyses within cross‐sectional designs are substantially biased (Maxwell & Cole, 2007), it is worthwhile exploring how these constructs may interact to predict behavioral dysregulation cross‐sectionally. Although tests of moderation involving continuous variables have inherently limited power (Cohen et al., 2013; Memon et al., 2019), it is paramount to continue to explore the interrelationships between brooding and negative urgency with behavioral dysregulation in a clinical sample. Without accounting for the role of negative urgency in behavioral dysregulation, the importance of rumination, including brooding and reflection, may be overvalued, particularly within the ECM. Consequently, understanding these interrelationships will refine knowledge about the development and maintenance of dysregulated behaviors and subsequent psychopathology, and potentially update knowledge pertaining to theoretical models such as the ECM. In turn, this will enhance the focus of relevant therapeutic approaches to appropriate targets, maximizing treatment outcomes and preventative efforts.

### 1.4 The present study

This study has been designed to address the issues argued previously: definitional ambiguity surrounding the form of rumination implicated in emotional cascades; and the historical omission of negative urgency when considering the role of rumination in dysregulated behaviors, and more broadly, in the ECM. Therefore, the aims of the present study are:

(1) to clarify the role of brooding in behavioral dysregulation, including when accounting for negative urgency, and
(2) to test the interrelationships between negative urgency and a relevant component of the ECM including brooding, with dysregulated behaviors.

Using SEM in a large clinical sample, the current study examined the relationship between the latent variables of Psychological Distress, Brooding, Negative Urgency, and Behavioral Dysregulation, which was comprised of alcohol use, drug use, binge eating and purging, and NSSI. The following were hypothesized when controlling for age and gender:

(H1) brooding, psychological distress, and negative urgency would be positively related to behavioral dysregulation;

(H2) negative urgency would moderate the relationship between brooding and behavioral dysregulation, such that individuals higher in both negative urgency and brooding would report higher behavioral dysregulation;

(H3) high brooding, psychological distress, and negative urgency would be significantly positively related to each of the indicators of behavioral dysregulation (i.e., NSSI, alcohol use, drug use, binge eating, and purging, respectively); and

(H4) negative urgency would moderate the relationship between brooding and each respective indicator of behavioral dysregulation, such that individuals higher in both negative urgency and brooding would report higher engagement in each respective dysregulated behavior.

## 2 METHOD

### 2.1 Participants

The participants were 385 young people (*M*_age_ = 20.45, SD = 2.47), who were accessing mental health and/or alcohol and other drug (AOD) services from four mental health and AOD treatment sites across Victoria, Australia (including two primary mental health programs, a residential drug rehabilitation program, and a drug and alcohol day program). Inclusion criteria involved being aged between 16 and 25 years old, currently accessing services at the relevant site, and providing consent to participate. Exclusion criteria included experiencing any acute mental health concerns preventing the young person from giving informed consent (e.g., a current psychotic episode, suicidality requiring immediate intervention). Sociodemographic characteristics of participants are presented in Table 1. The characteristics of this sample are consistent with previous studies (Lubman et al., 2016; Mitchell et al., 2016), and demonstrate the typical psychosocial complexity of this population.

**Table 1:** Demographic and social characteristics of participants.

	*n*	*%*
Gender		
Female	240	62
Male	84	22
Nonbinary	61	16
Aboriginal and/or Torres Strait Islander	12	3
Identifies as LGBTIQA+	228	59
Questioning	44	11
Born in Australia	338	88
Language other than English spoken at home	19	5
Did not complete high school	92	25
Unemployed	210	56
Diagnosed with or treated for an MH condition	287	78
Diagnosed with or treated for a PH condition	135	36
Experienced family violence	249	67
CPS involvement during childhood	68	18
Attended court for a criminal matter	50	13.5
Slept rough or couch surfed during lifetime	194	52
Slept rough, couch surfed, or in crisis accommodation last night	34	9
Experienced homelessness before age 18	66	51

Abbreviations: CPS, Child Protective Services; LGBTIQA+, lesbian, gay, bisexual, transgender, intersex, queer, asexual, and others; MH, mental health; PH, physical health.

### 2.2 Procedure

The current study received ethics approval from the host university and the involved institution's human ethics committees. Written informed consent was provided by participants at the start of the online survey. A convenience sampling strategy was employed by displaying flyers and posters across the four treatment sites to advertise the study to young people attending the service. These individuals were invited by researchers to take part in the anonymous online survey and were informed that participation was entirely voluntary and would not impact their access to the relevant service. The survey, which took approximately 20 minutes to complete, was administered via a tablet, on‐site computer, or on participants' own devices. If required, participants were assisted with the completion of the survey by a member of the research team or clinical staff (e.g., if the young person had a low literacy level). Following completion of the survey, participants were reimbursed with a $15 supermarket voucher.

### 2.3 Measures

Measures in the current study were all in a self‐report format and were selected following careful consideration of participants' standard literacy levels. Where possible, brief measures with demonstrated reliability and validity were utilized to minimize participant burden.

#### 2.3.1 Rumination: Brooding and reflection

The shortened version of the original *Ruminative Response Scale* (RRS; Nolen‐Hoeksema & Morrow, 1991) is a 10‐item scale, comprised of two subscales, that captures both adaptive and maladaptive forms of rumination, brooding and reflection (Treynor et al., 2003). The RRS is the most widely used measure of rumination, and the shortened version has demonstrated good reliability and concurrent validity (Erdur‐Baker & Bugay, 2010). Items from the brooding and reflection subscales were used to create a latent variable of brooding and reflection, respectively, with each being tested individually across separate models. These subscales demonstrated good and acceptable internal reliability; brooding (*α* = 0.83) and reflection (*α* = 0.77).

#### 2.3.2 Negative urgency

The *SUPPS‐P Impulsive Behavior Scale* (Lynam, 2013) is a shortened version of the original UPPS‐P scale (Whiteside & Lynam, 2001). The four items from the negative urgency subscale, which measures the tendency to behave impulsively in response to negative affect, were used in the current study to create the negative urgency latent variable. Higher values indicate an increased tendency to behave impulsively in response to negative affect. The subscale has demonstrated good reliability as a shorter alternative to the original UPPS scale (Cyders et al., 2014), and has been validated in samples with adolescents (d'Acremont & Van der Linden, 2005). This subscale demonstrated good internal reliability in the current study (*α* = 0.84).

#### 2.3.3 Psychological distress

The *Depression, Anxiety and Stress Scales* (DASS‐21; Lovibond & Lovibond, 1995) is a 21‐item scale that is used to detect psychological distress, via the presence of depressive, anxiety, and stress symptoms. This shortened version of the original DASS‐42 has shown good factorial validity in samples of young people (Willemsen et al., 2011). The scale is comprised of three subscales respectively measuring depression, stress, and anxiety symptoms, where each item response is scored from 0 (*Did not apply to me at all)* to 3 (*Applied to me very much*). Given recent findings from Shaw et al. (2017) that the DASS‐21 fails to discriminate between these three states in adolescents and instead is a reliable indicator of general distress, the three subscales were used to create a latent variable of psychological distress. Each of these subscales demonstrated good to excellent internal reliability: anxiety (*α* = 0.86), stress (*α* = 0.86), and depression (*α* = 0.91).

#### 2.3.4 Behavioral dysregulation

##### 2.3.4.1 NSSI

To minimize participant burden, a brief scale was adapted from the format of the Timeline Follow‐back measure (Sobell & Sobell, 1992) and developed by the authors to examine the frequency of engagement in NSSI. This item required participants to select based on a calendar, the number of days they engaged in NSSI in the past month. NSSI was defined to participants as acts of self‐injury that involved the intention to hurt oneself, but in the absence of wanting to end their life (e.g., cutting or burning). A binary variable was then created and attributed a score of 1 if participants endorsed a frequency greater than 0 for the entire month.

##### 2.3.4.2 Binge eating and purging

The *Eating Disorder Diagnostic Scale* (EDDS; Stice et al., 2000) is a 22‐item questionnaire measuring anorexia nervosa (AN), bulimia nervosa (BN), and binge eating disorder (BED) based on the DSM‐IV (American Psychiatric Association, 1994) criteria. It consists of a combination of Likert, dichotomous and frequency scores, and open‐ended questions about weight and height. For the present study, only four items were utilized (Items 5, 6, 15, and 16), to capture frequencies of binge and purging behaviors. Regarding binge eating, a binary variable was created and was attributed a score of 1 if participants answered yes to both eating an objectively large amount of food and experiencing a loss of control while eating. For purging, a binary variable was created and was attributed a score of 1 if participants endorsed a frequency greater than 0 on Items 15 (made yourself vomit) and 16 (used laxatives).

##### 2.3.4.3 Alcohol use

The *Alcohol Use Disorders Identification Test* (AUDIT; Babor & Grant, 1989; Babor et al., 1992; Saunders et al., 1993) is a 10‐item self‐report measure used to identify individuals with current difficulties with alcohol use. It offers an assessment of frequency, problems, and symptoms of alcohol dependency, and has demonstrated consistent reliability and validity as an alcohol screening measure in primary care settings (de Meneses‐Gaya et al., 2009; Fiellin et al., 2000). For the current study, the total score was used. This scale demonstrated good internal reliability (*α* = 0.87).

##### 2.3.4.4 Drug use

The *Drug Use Disorders Identification Test* (DUDIT; Berman et al., 2003) is an 11‐item self‐report measure developed for the identification of problematic substance use and is analogous to the AUDIT. It offers an assessment of frequency, problems, and symptoms of dependency in relation to substance use, and has good internal consistency and test−retest reliability (Matuszka et al., 2014). It has been validated in clinical samples of substance users in outpatient or residential services (Hildebrand, 2015), and in young people (Hillege et al., 2010). The total score of the DUDIT was used. This scale demonstrated excellent internal reliability (*α* = 0.92).

### 2.4 Statistical analysis

Statistical analyses were conducted in Stata v13.0 and MPlus v8.1.5. Given the anonymous nature of the survey which was administered online, a requirement of individuals' responses was that they had completed at least the first demographic item at the beginning of the survey to be considered a participant in the study. Additionally, 27 participants were excluded as they had completed the survey within 5 min, based on Huang et al. (2012) response time criteria for careless responding. Beyond this, 57 participants were found to have incomplete data across measures used in the current study. This final data set had a monotonic pattern of missingness due to participants not being able to recommence the survey once they discontinued. These data were missing at random; therefore, full information maximum likelihood estimation was used to account for missing data due to incomplete response (Enders, 2010).

To answer the primary research questions, SEM was used. First, a series of latent variables were estimated in separate models using confirmatory factor analysis; one for Psychological Distress, Negative Urgency, Brooding, Reflection, and Behavioral Dysregulation. The indicators used for each latent variable are described in later sections. Latent variables were scaled by fixing factor variances to 1. A measurement model was then estimated in which these latent factors were estimated as correlated factors in a single model. Once good fitting latent variables were estimated, a structural model was specified in which the latent Behavioral Dysregulation variable was regressed to Psychological Distress, Brooding, Negative Urgency, and the covariates of age and gender. In a separate model, it was examined whether the relationship between Brooding and Behavioral Dysregulation was moderated by Negative Urgency. The same relationship above was estimated for each Behavioral Dysregulation variable, separately, to explore whether the hypothesized relationships may be specific to a particular behavior. To test the specificity of the model to brooding, a separate model including Reflection in place of Brooding was subsequently run, with all other variables remaining the same (see Supplementary Materials).

Model fit was assessed using *χ* ^2^ statistic, which assesses the discrepancy between the actual data and the hypothesized model. While a significant *χ* ^2^ represents a lack of fit (Hu & Bentler, 1999), it can also be sensitive to large sample sizes (Tabachnick et al., 2007). Therefore, additional fit indices (i.e., comparative fit index [CFI] and the root‐mean square error of approximation [RMSEA]) were used to test the model fit. Standard cut‐off criteria for good fit were used to judge the model as a whole and consisted of CFI values greater than 0.95, and RMSEA values less than 0.06. Robust maximum likelihood estimation was employed across all models. Moderation analyses were specified using the XWITH latent variable interaction command in MPlus (Muthén & Muthén, 2006). Statistically significant latent variable interactions were explored using simple slope analyses (Muthén & Asparouhov, 2003).

## 3 RESULTS

### 3.1 Preliminary analyses

The pairwise correlations, means, and standard deviations for the variables used in the current study are presented in Table 2. Item‐level correlations of the RRS and SUPPS‐P are provided given these items were factors for latent variables of brooding, reflection, and negative urgency, respectively. As illustrated, items planned for factor analyses were significantly positively intercorrelated, including the DASS‐21 subscales, SUPPS‐P negative urgency items, and RRS items. The measures of individual dysregulated behaviors were also significantly positively intercorrelated, except for the relationships between alcohol use and binge eating, and NSSI, respectively. The means for measures of the DASS‐21 subscales, alcohol use, and drug use are substantially higher than those observed in a normative sample (Henry & Crawford, 2005), and exceeded previously observed means in clinical samples (Antony et al., 1998; de Meneses‐Gaya et al., 2009), demonstrating the level of distress and dysregulation within this particular sample. Considering individuals' engagement in dysregulated behaviors, 69% reported engaging in NSSI during their lifetime (*n* = 240), and of those, 31% reported engaging in NSSI in the past month (*n* = 74). Seventy‐four percent had drunk alcohol in the past year (*n* = 267), and of those, 81% had drunk alcohol in the past month (*n* = 215). Fifty‐one percent had used drugs other than alcohol during the past year (*n* = 179), and of those, 70% had used drugs in the past month (*n* = 126). Twenty‐nine percent of the sample reported binge eating in the past 3 months (*n* = 101). Sixteen percent of the sample reported purging (or using laxatives) in the past 3 months (*n* = 55).

**Table 2:** Pairwise correlations, means, and standardized deviations for included variables.

	1	2	3	4	5	6	7	8	9	10	11	12	13	14	15	16	17	18	19	20	21	22	23	24
1. Age	—																							
2. Gender	0.08	—																						
3. DASS_S	0.11*	0.13*	—																					
4. DASS_A	0.00	0.07	0.80***	—																				
5. DASS_D	0.05	0.20***	0.63***	0.61***	—																			
6. RRS_1	−0.05	0.08	0.40***	0.48***	0.37***	—																		
7. RRS_3	0.11*	0.08	0.49***	0.47***	0.31***	0.49***	—																	
8. RRS_6	0.07	0.09	0.46***	0.43***	0.37***	0.37***	0.51***	—																
9. RRS_7	−0.04	0.15**	0.39***	0.48***	0.38***	0.57***	0.45***	0.44***	—															
10. RRS_8	0.05	0.13*	0.48***	0.52***	0.46***	0.47***	0.54***	0.53***	0.62***	—														
11. RRS_2	0.13*	0.12*	0.40***	0.39***	0.30***	0.44***	0.63***	0.46***	0.47***	0.45***	—													
12. RRS_4	0.06	0.06	0.32***	0.33***	0.25***	0.40***	0.56***	0.44***	0.43***	0.45***	0.56***	—												
13. RRS_5	0.05	0.08	0.13*	0.21***	0.11*	0.22***	0.19***	0.12*	0.15**	0.15**	0.20***	0.29***	—											
14. RRS_9	0.15**	0.18***	0.44***	0.47***	0.43***	0.34***	0.49***	0.44***	0.37***	0.53***	0.56***	0.43***	0.26***	—										
15. RRS_10	0.04	0.06	0.27***	0.32***	0.27***	0.38***	0.35***	0.33***	0.32***	0.33***	0.40***	0.61***	0.29***	0.38***	—									
16. SUPPS‐P_6	0.06	0.04	0.29***	0.26***	0.22***	0.12*	0.19***	0.30***	0.18**	0.25***	0.13*	0.21***	0.05	0.19***	0.23***	—								
17. SUPPS‐P_8	0.04	0.05	0.34***	0.35***	0.27***	0.20***	0.28***	0.35***	0.25***	0.38***	0.22***	0.29***	0.05	0.28***	0.23***	0.60***	—							
18. SUPPS‐P_13	−0.08	0.02	0.35***	0.30***	0.23***	0.17**	0.22***	0.29***	0.25***	0.31***	0.10	0.23***	0.10	0.25***	0.20***	0.58***	0.61***	—						
19. SUPPS‐P_15	0.11*	0.05	0.31***	0.21***	0.14*	0.16**	0.28***	0.25***	0.29***	0.31***	0.26***	0.25***	0.12*	0.24***	0.21***	0.50***	0.51***	0.60***	—					
20. Alcohol use	−0.01	−0.19***	0.14**	0.15**	0.08	0.09	0.07	0.21***	0.13*	0.17**	0.07	0.09	0.01	0.10	0.12*	0.22***	0.23***	0.23***	0.23***	—				
21. Drug use	−0.01	−0.09	0.16**	0.17**	0.06	0.10	0.12*	0.19***	0.10	0.15**	0.13*	0.14*	−0.02	0.13*	0.10	0.32***	0.25***	0.31***	0.30***	0.42***	—			
22. Purging	−0.10	0.14**	0.22***	0.25***	0.23***	0.16*	0.09	0.08	0.15**	0.13*	0.07	0.12*	0.16**	0.12*	0.20***	0.12*	0.17**	0.22***	0.12*	0.18***	0.16**	—		
23. Binge eating	0.03	0.20***	0.21***	0.28***	0.25***	0.17**	0.18	0.24***	0.27***	0.24***	0.20***	0.017**	0.05	0.21***	0.16**	0.20***	0.24***	0.25***	0.22***	0.18	0.25***	0.38***	—	
24. NSSI	−0.05	0.13*	0.22***	0.22***	0.28***	0.18***	0.15**	0.14*	0.21***	0.19***	0.12*	0.13*	0.03	0.11*	0.17**	0.24***	0.16**	0.22***	0.18**	0.09	0.13*	0.24***	0.14*	—
Mean	20.45	—	22.80	18.21	24.14	1.33	1.54	1.88	1.50	1.83	1.65	1.53	0.89	1.63	1.45	2.48	2.48	2.50	2.41	7.49	7.10	—	—	—
SD	2.47	—	9.55	10.20	11.01	0.97	0.98	0.92	0.99	0.96	0.93	0.98	0.93	1.04	0.95	1.01	1.06	1.02	1.06	7.70	10.73	—	—	—

Abbreviations: DASS_A, DASS‐21 Anxiety subscale; DASS_D, DASS‐21 Depression subscale; DASS_S, DASS‐21 Stress subscale; NSSI, non‐suicidal self‐injury; RRS, Ruminative Response Scale; SUPPS‐P, Shortened UPPS‐P.

* *p* < 0.05;

** *p* < 0.01;

*** *p* < 0.001

### 3.2 Measurement model analyses

Preliminary measurement analyses were first conducted to test whether the measured variables fit well together when forming the hypothesized latent variables. Table S1 presents the model fit when estimating each latent variable separately. The Psychological Distress latent variable was comprised of three variables, including the three subscales of the DASS‐21 (Depression, Anxiety, and Stress). The Negative Urgency latent variable was comprised of four items from the SUPPS‐P Negative Urgency subscale. The Brooding latent variable was comprised of five items from the RRS brooding subscale, with the Reflection latent variable being comprised of five items from the RRS reflection subscale. All factors were found to have a good fit when estimated separately. All indicators for these latent variables were also found to have significant factor loadings onto their respective latent variable. The Behavioral Dysregulation latent variable was also developed and comprised of alcohol use, drug use, binge eating, purging, and NSSI. After estimating residual correlations between the alcohol and drug use indicators, and binge eating and purging indicators, this model had a good fit.

The standardized factor loadings of each latent variable on its corresponding observed variables, and correlational paths between latent variables which were included in the main analyses, are displayed in the correlated factors model in Figure S1. This model had acceptable fit, *χ* ^2^(111, *n* = 361) = 200.95, *p* < 0*.*001, CFI = 0.95, RMSEA = 0.05. All indicators had high loadings on their respective variables. For behavioral dysregulation, all five variables had significant factor loadings onto the latent variable, with binge eating having the highest (*λ* = 0.47), and alcohol use having the lowest (*λ* = 0.36).

### 3.3 Structural model with behavioral dysregulation latent variable as the outcome

**Figure 1:**
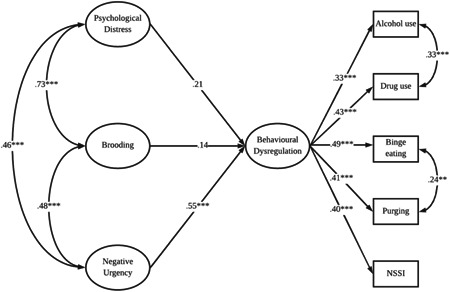
Standardized factor loadings and regression coefficients for structural model demonstrating the relationship between psychological distress, brooding, and negative urgency on behavioral dysregulation, adjusting for age and gender. NSSI, nonsuicidal self‐injury; *p < 0.05; **p < 0.01; ***p < 0.001.

Addressing H1, Figure 1 presents the regression of the Behavioral Dysregulation latent variable on Psychological Distress, Brooding, Negative Urgency, and covariates. This model exhibited acceptable fit, *χ* ^2^(159, *n* = 361) = 323.17, *p* < 0.001, CFI = 0.92, RMSEA = 0.05. Only the path between Negative Urgency and Behavioral Dysregulation was significant (*p* < 0.001), which demonstrated a moderate effect (*β* = 0.55, 95% CI [0.33, 0.76]). In a separate model addressing H2, there was no evidence that Negative Urgency moderated the relationship between Brooding and Behavioral Dysregulation latent variables (*β* = 0.09, *p* = 0.721, 95% CI [−0.42, 0.60]). The same pattern of results occurred when Reflection was entered in the model, in place of Brooding (see Supplementary Materials).

### 3.4 Structural models with each dysregulated behavior as outcomes

To test H3 and H4 regarding the ECM for individual dysregulated behaviors, five separate SEM analyses were run. All included analyses adjusted for age and gender. Standardized beta weights and confidence intervals for main effects are provided in Table 3. Negative urgency significantly and positively predicted each individual behavior, with weak to moderate effects. Psychological distress significantly and positively predicted purging, with a moderate effect. No other relationships were found between Psychological Distress, Negative Urgency, and individual dysregulated behaviors.

**Table 3:** Standardized beta weights with confidence intervals for latent variables predicting each dysregulated behavior.

	Individual dysregulated behavior				
	Alcohol use	Drug use	Binge eating	Purging	NSSI
Predictor	*β*	*β*	*β*	*β*	*β*
	95% CI	95% CI	95% CI	95% CI	95% CI
	[LL, UL]	[LL, UL]	[LL, UL]	[LL, UL]	[LL, UL]
PD	−0.01 [−0.18, 0.17]	−0.03 [−0.20, 0.15]	0.08 [−0.15, 0.30]	0.42** [0.16, 0.69]	0.19 [−0.06, 0.43]

Brooding	0.10 [−0.10, 0.30]	0.06 [−0.13, 0.25]	0.20 [−0.02, 0.42]	−0.20 [−0.48, 0.08]	0.09 [−0.20, 0.37]

NU	0.26*** [0.12, 0.40]	0.38*** [0.24, 0.53]	0.26** [0.11, 0.41]	0.22* [0.01, 0.44]	0.24** [0.07, 0.42]


Abbreviations: NSSI, nonsuicidal self‐injury; NU, negative urgency; PD, psychological distress.

* *p* < 0.05

** *p* < 0.01

*** *p* < 0.001.

### 3.5 Moderation analyses

In separate models, interactions of Negative Urgency and Brooding were specified on each behavior to address H4. The same findings generally occurred for individual dysregulated behaviors as for the overall model, with no evidence of Negative Urgency moderating the relationship between Brooding and drug use (*β* = 0.16, *p* = 0.566), binge eating (*β* = −0.09, *p* = 0.549), NSSI (*β* = −0.04, *p* = 0.813) alcohol use (*β* = 0.23, *p* = 0.072), or purging (*β* = 0.11, *p* = 0.430), respectively. The same pattern of results occurred when Reflection was entered in the models, in place of Brooding.

## 4 DISCUSSION

Using SEM techniques, this study aimed to explore the role of brooding, a key component of the ECM, alongside negative urgency, while utilizing a clinical sample of treatment‐seeking young adults who engaged in various dysregulated behaviors. The inclusion of negative urgency alongside brooding and distress, including examining the interrelationships between negative urgency and brooding with dysregulated behaviors, intended to further the theoretical understanding of key components of the ECM, and potentially reveal whether negative urgency should be included in future applications of the ECM. Contrary to H1, and to previous findings from the ECM more broadly, only negative urgency, and not distress or brooding, was significantly positively related to overall behavioral dysregulation. Though not a core focus for the current study, these results also did not differ when reflection was included in the model, instead of brooding. H2 was also unsupported, with no evidence of negative urgency moderating the relationship between brooding and overall behavioral dysregulation. These results also did not differ for reflection, as negative urgency did not significantly moderate the relationship between reflection and overall behavioral dysregulation. Findings for separate models examining H3 and H4 mirrored those of the overall model. There was only partial support for H3, with only negative urgency (not brooding or distress), being significantly positively related to each dysregulated behavior (NSSI, alcohol use, drug use, binge eating, and purging). Contrary to H4, negative urgency did not moderate the relationship between brooding and any of the dysregulated behaviors. Given the limited studies on the ECM within clinical cohorts, the results of the present study are novel and demonstrate that the components of the ECM examined (distress and brooding) may not be applicable to this particular clinical sample of treatment‐seeking young adults. Instead, the present study has provided further support for the relationship between negative urgency and behavioral dysregulation, even when controlling for psychological distress. These findings support the broad literature which concludes that deficits in emotion regulation contribute to such behaviors (Aldao & Nolen‐Hoeksema, 2010; Berg et al., 2015).

### 4.1 Findings in the context of the broader literature on the ECM

The results from the current study were inconsistent with the existing literature examining rumination in the context of emotional cascades utilizing SEM techniques (Gardner et al., 2014; Jungmann et al., 2016; Selby et al., 2008, 2009; Tuna & Bozo, 2014), finding no evidence that high levels of brooding and psychological distress were associated with increased behavioral dysregulation in this sample. While these iterations of the ECM included different measures of rumination to the current study (e.g., Cognitive Emotion Regulation Questionnaire; CERQ; Garnefski et al., 2001), additional studies which have utilized brooding in conjunction with other measures of rumination have found evidence supporting the ECM in community and forensic samples (Gardner et al., 2014; Jungmann et al., 2016; Selby et al., 2009). In fact, brooding had consistently been demonstrated to be one of the most strongly related indicators of the rumination latent variable observed in these studies, if not the strongest (Gardner et al., 2014; Selby et al., 2009).

The differences observed in the present sample when compared to Selby et al. (2009) and Gardner et al. (2014) may be due to a number of factors. Given the current focus on specifying brooding's role in emotional cascades and subsequent behavioral dysregulation, it is possible that brooding alone does not appropriately capture the intrinsic processes that occur during an emotional cascade. Brooding refers to the passive focus on depressive symptoms and their causes, and a comparison of one's current situation with an unachieved standard (Treynor et al., 2003). This definition could conflict with the actual cognitive processes occurring within this sample, as participants who engaged in dysregulated behaviors may not be focusing specifically on exact causes for their low mood or perceived shortcomings, but rather engaging in persistent negative, repetitive and/or intrusive thoughts, self‐appraisals, or evaluations about interpersonal situations or past events, including memories of conflict or trauma (Lyubomirsky & Nolen‐Hoeksema, 1995; Lyubomirsky et al., 1999). Recent qualitative research has similarly characterized this cohort's ruminative pattern as generalized persistent negative, repetitive thought (Sloan et al., 2020). While the current study chose the measure of brooding in the context of its wide prior use and ability to distinguish adaptive from maladaptive rumination, it may be possible that alternative rumination measures are required to demonstrate emotional cascades in this sample, such as the Anger Rumination Scale (ARS; Sukhodolsky et al., 2001).

Additionally, the heightened rates of distress, dysregulation, and psychosocial vulnerabilities of the current sample may explain the nonsignificant relationships between brooding and behavioral dysregulation, and between distress and behavioral dysregulation. As noted, the means observed within this sample, particularly for scales measuring psychological distress, were extremely high; in fact, they were at least double those observed in normative samples (Henry & Crawford, 2005). In comparison, historical studies on the ECM reported means of psychological distress, brooding, and various dysregulated behaviors that were only slightly elevated or even similar to normative samples (Selby et al., 2008, 2009). Due to the level of widespread high reporting of distress within this sample, there may be ceiling effects occurring, which precludes the current study from identifying significant relationships among those high in brooding but lower in psychological distress, and less frequent episodes of behavioral dysregulation. Given this sample endorsed high levels of distress but did not demonstrate a significant relationship between brooding and behavioral dysregulation, perhaps the integral relationships between brooding, distress, and dysregulated behaviors that underlie the ECM only apply to individuals with low levels of these symptoms. Future studies more broadly exploring the ECM within a clinical, treatment‐seeking sample may benefit by including a comparative community sample, or recruiting participants according to low, moderate, and high distress to ensure all groups are represented. Furthermore, the average level of brooding reported in our study was in fact lower than comparable samples from previous studies exploring the ECM (Gardner et al., 2014), despite the abovementioned high rates of psychological distress. This creates further uncertainty over the role of brooding in the ECM, given such high levels of distress and frequency of dysregulated behaviors reported within the current sample.

### 4.2 Negative urgency as a potential addition to the ECM

To our knowledge, the current study is the first to examine the relationship between negative urgency and dysregulated behaviors alongside brooding and psychological distress, as well as being the first to consider negative urgency's role as a moderator in the relationship between brooding and behavioral dysregulation. The results from the current study provoke further thought and consideration for the role of negative urgency within the ECM. The results confirmed that individuals high in negative urgency engaged in higher levels of all forms of behavioral dysregulation, including alcohol use, drug use, binge eating, purging, and NSSI, even when controlling for psychological distress, gender, and age. These findings evidenced within a clinical sample provide strong support for the UPPS‐P model, demonstrating the difficulties this population encounters in managing psychological distress and regulating emotions effectively. While no published studies on the ECM included negative urgency as a predictor variable, urgency had been incorporated into the model in two studies, as an indicator of the latent outcome variable, behavioral dysregulation (Jungmann et al., 2016; Selby et al., 2008). In both studies, urgency was the strongest indicator of behavioral dysregulation, with markedly stronger factor loadings than actual dysregulated behaviors. Compared to these two initial studies, it is likely that the difference in the current findings is due to the conceptualization of negative urgency as a potential vulnerability rather than a behavioral outcome. This reinforces the UPPS‐P model proposed by Whiteside and Lynam (2001) and supports findings described by Berg et al. (2015), that regardless of the type of dysregulated behavior, all share an intrinsic drive to reduce psychological distress. While H1 and H3 were unsupported with regard to brooding being implicated in behavioral dysregulation, this may help explain the pattern of dysregulation within the current sample. Perhaps, rather than intentionally trying to distract themselves from cognitive processes occurring, these individuals lack the appropriate problem‐solving skills and emotional capacity to identify and address their current levels of distress. This could be particularly relevant for a population of young people seeking treatment, given their stage of neurobiological development (Steinberg, 2008) where brain structures such as the prefrontal cortex are still maturing, which are responsible for higher‐order processes such as planning, judgment, and decision‐making skills (Labouvie‐Vief, 2006). Coupled with emotion dysregulation, optimal decision‐making skills are likely to be compromised and replaced with more immediate, sensory‐based forms of distraction, such as dysregulated behaviors (Beauchaine et al., 2007; Blakemore & Choudhury, 2006; van Toor et al., 2011). This has important implications for prevention and intervention for young people experiencing a range of psychopathology symptoms mentioned above. Future studies should attempt to again explore these interrelationships to ascertain whether negative urgency is in fact the determining factor for behavioral dysregulation, or whether brooding and psychological distress can also account for why an individual may be likely to engage in these behaviors.

Contrary to H2, negative urgency did not moderate the relationship between brooding and behavioral dysregulation. Research examining these constructs together is only in its early stages (Valderrama et al., 2016; Wang & Borders, 2018), and thus the current study contributes important information toward the understanding of these interrelationships. Interestingly, previous studies exploring these interrelationships with regard to suicide risk (Valderrama et al., 2016) and disordered eating (Wang & Borders, 2018) have reported mixed results. Significant indirect effects were found for rumination on disordered eating via negative urgency, though this was significant in the nonclinical sample only, and did not remain significant at follow‐up (Wang & Borders, 2018). Valderrama et al. (2016) found no evidence of a significant indirect effect of rumination on suicide risk through negative urgency. While the implications from Valderrama et al. (2016) supported evidence of a cognitive model of suicidal behavior and risk (Wenzel & Beck, 2008), the current study's clinical sample emphasizes the importance of impulsivity through the strong association between negative urgency and various dysregulated behaviors. Despite using a different statistical approach to Valderrama et al. (2016) and Wang and Borders (2018), the current study provides important collateral information on the nature of these interrelationships. While the current study focussed on brooding only, Wang and Borders (2018) also accounted for anger rumination, which was found to be indirectly related to disordered eating through negative urgency. In light of Wang and Borders (2018) study, together with the current findings, it is possible that the depressive‐focused conceptualization of brooding does not fully capture processes associated with behavioral dysregulation in a clinical sample. Furthermore, these findings also suggest that these components of the ECM may only apply to individuals with symptoms of low severity, akin to nonclinical samples which have previously demonstrated support for the ECM (Selby et al., 2008, 2009; Tuna & Bozo, 2014).

### 4.3 Examining individual dysregulated behaviors

As noted in H3 and H4, the current study also sought to test the model among individual behaviors. When examining the relationship between brooding and each dysregulated behavior, results indicated that brooding was not significantly related to increased engagement in any individual form of dysregulated behavior, and while not a core focus for the current study, these results also did not differ for reflection. The current study adds to the limited findings from the literature exploring these rumination subtypes in various symptoms of psychopathology (Devynck et al., 2017; Gordon et al., 2012; Hoff & Muehlenkamp, 2009; Willem et al., 2011). To our knowledge, the present study is the first to explore the relationship between brooding and purging, and while there was no evidence of a significant association, this is a unique contribution to the scant literature examining this relationship (Meaney et al., 2016). In comparison, this study adds to the mixed findings between brooding and substance use (Willem et al., 2011, 2014), and thus generates additional doubt over the nature of this relationship. Future research should consider re‐examining these subtypes alongside negative urgency, while considering distinguishing substance use from substance dependence and/or problems.

Regarding NSSI, the current findings are consistent with Zaki et al. (2013), who also failed to find a significant relationship between brooding and NSSI. Instead, they found that the interaction of brooding together with emotional differentiation, significantly predicted NSSI frequency. This demonstrates the need to consider additional factors alongside brooding in predicting such behaviors. Similarly, this appears relevant for the relationship between brooding and binge eating, which was nonsignificant in the current study; however, brooding has previously been found to significantly interact with body dissatisfaction to predict binge eating (Gordon et al., 2012). Consistent with Devynck et al. (2017), Hoff and Muehlenkamp (2009) and Willem et al. (2011), these findings suggest that a direct relationship between brooding and various dysregulated behaviors is difficult to ascertain. Instead, additional variables may be required to fully conceptualize this phenomenon, although the variables used to supplement this understanding will likely depend on the nature of the dysregulated behavior.

### 4.4 Implications

The present study is the first to explore components of the ECM in a clinical sample of young people exhibiting various dysregulated behaviors concurrently, and while it is difficult to generalize the overall applicability of the model (or lack of) to this cohort based on the current findings alone, it does provide much‐needed data about this population which had not been represented previously in the ECM literature. The present sample displayed significantly heightened levels of distress, frequent behavioral dysregulation, and high rates of psychosocial vulnerabilities, compared to those observed by Selby et al. (2008), Tuna and Bozo (2014), and Jungmann et al. (2016), which may explain the current mixed findings. These disparities highlight the distinct differences that clinical, treatment‐seeking samples with high levels of distress and simultaneous dysregulated behaviors encounter, and thus necessitates ongoing exploration and representation within the literature. As third‐wave therapies quickly evolve, with a growing number of specific rumination‐focused modalities used to treat varying psychopathology (Ruiz et al., 2016; Watkins, 2018), the current study provides a crucial examination of the relationship between rumination and behavioral dysregulation. With no evidence of a significant relationship in the current sample, the utility in addressing rumination in the treatment and prevention of behavioral dysregulation remains uncertain. Given the emphasis within third‐wave therapies on cognitive processes over and above symptomology, such modalities require more research on these aforementioned processes, to determine whether they may contribute to behavioral dysregulation, and thus further inform whether targeted rumination interventions may be effective in reducing these behaviors.

The current study has implications for the significance of the role of negative urgency in behavioral dysregulation. While generalizations for individual behaviors are limited, results from the overall model indicated that those higher in negative urgency were more likely to become behaviorally dysregulated, even when psychological distress was accounted for. This has important implications for prevention, assessment, and psychological interventions specific to these dysregulated behaviors. Preventative efforts may be focused on increasing community understanding about personality vulnerabilities implicated in behavioral dysregulation, particularly during critical phases of development such as adolescence, when the risk for incidence of mental health difficulties and risk‐taking behavior is especially heightened (Kessler et al., 2005). Additionally, enquiring and screening for individuals who exhibit higher levels of negative urgency during intake assessments will aid in a more comprehensive understanding of their risk profile, assist in screening for co‐occurring dysregulated behaviors, and aid in determining the most appropriate therapeutic approach for the client. Such therapeutic approaches may benefit these individuals with emotional experiential difficulties, by focusing on a broad array of skills, such as developing capacity for problem‐solving, and increasing an individual's ability to be mindful of emotions and current impulses. Specific modalities, such as Dialectical Behavior Therapy (Linehan, 1993), target negative urgency by building skills in tolerating distress and resisting urges to engage in unhelpful behaviors, and thus are likely to be of great benefit for this population. Additionally, interventions informed by impulsivity and negative urgency, which operate by targeting decision‐making abilities to remain focused on goals and values despite strong emotions (Bowen et al., 2021; Hall et al., 2021; Morton & Shaw, 2012; Nezu et al., 2012; Sloan et al., 2018), should also be considered for treatment of these behaviors.

### 4.5 Limitations and future directions

Interpretation of results from the current study should be considered in light of a number of key limitations. Most notably, the cross‐sectional, correlational design is unable to substantiate any causal claims about the relationship between negative urgency and behavioral dysregulation. The between‐person analyses employed in the current study also limit the extent to which generalizations can be made regarding the within‐person processes of the ECM (Fisher et al., 2018). Given the limited explorations of the ECM in clinical, treatment‐seeking samples, the current study provides a meaningful contribution to the scant literature; however, future studies should endeavor to examine within‐person effects regarding the ECM, in a clinically representative sample. Moreover, as the chosen measures of psychological distress specify the past 2‐week period, while brooding and negative urgency utilize more trait‐like measures, and the behavioral dysregulation variables have varying time periods, temporal discrepancies within these relationships are possible. This may also account for the hypotheses not being supported, given participants' levels of distress and brooding were not measured immediately before behavioral dysregulation. Given the characteristics of the current sample, which included individuals seeking both short‐ and longer‐term treatment in outpatient and residential services, a cross‐sectional, self‐report design was utilized for minimal participant burden. However, where feasible, future studies exploring these relationships in clinical samples should utilize EMA methodology with a focus on state‐based measures, to provide further understanding of the temporal nature of these relationships.

Considering the unique contribution this study provides with the inclusion of a clinical treatment‐seeking sample, some potential confounding factors arising from the sample should be noted. Firstly, the self‐report nature of the study inherently includes a degree of bias in participant responses; therefore, future clinical research may benefit from a combination of self‐report, as well as clinician and/or parent‐rated measures to add to the robustness and validity of these constructs. Notably, a majority of the sample identified as being LGBTIQA+, and while a significant portion of studies fails to capture these demographic data, the prevalence within this sample is much higher compared to population‐based studies (Pesola et al., 2014). These rates are however consistent with a previous study in a similar sample (Sloan et al., 2019). Subsequently, it is difficult to ascertain whether the findings observed in the current study are typical of a clinical sample, or attributable to the unique and additional stressors that the LGBTIQA+ population encounters. Future research may benefit from controlling for sexual minority in addition to gender and age.

The use of a number of measures to capture engagement in behavioral dysregulation should be noted. This is particularly pertaining to the measurement of binge eating, purging, and NSSI within this sample, which were treated as dichotomous variables, signifying either the presence or absence of having engaged in the specified behavior during the given time period. As such, this reduces much of the variability and information within the data, whereby statistical power to detect positive relationships is significantly reduced (Altman & Royston, 2006). This was an unavoidable limitation given the smaller subset within the sample which reported engaging in these behaviors. It is worth noting that previous studies on the ECM have treated measurements of NSSI the same, and still found evidence in support of the model. Furthermore, the measurement of alcohol use and other substance use relied on total scores based on the AUDIT and DUDIT, which also measures alcohol and drug‐related problems and thus spans broader conceptualizations than just dysregulated behaviors. Given the limited opportunity within the survey and the sample to adequately assess whether alcohol and substance use occurred in direct response to distress arising from brooding, this was deemed an unavoidable limitation. Similarly, other studies related to the ECM have measured alcohol use (and misuse) without specifying whether it occurred in relation to distress (Selby & Joiner, 2013, 2020).

Lastly, while exceeding guidelines based on Monte Carlo simulations (Wolf et al., 2013), the sample size was modest in comparison with previous SEM studies exploring the ECM. This was reinforced within the subgroup sample sizes for individual dysregulated behaviors, which may limit the extent to which these findings can be generalized across a similar sample. While the main effects findings pertaining to negative urgency are consistent with the existing literature, future studies exploring the same relationships should utilize screening to ensure the sample is representative of those who engage frequently in dysregulated behaviors.

### 4.6 Conclusion

The current study sought to test the relevance of key components of the ECM (brooding rumination and psychological distress) to a clinical treatment‐seeking sample of young people, while also considering the crucial role of negative urgency in behavioral dysregulation, in attempts to progress theoretical understandings of the ECM. Contrary to hypotheses, only negative urgency, and not psychological distress or brooding, was significantly positively related to behavioral dysregulation. With the exception of psychological distress being significantly positively related to purging, these same findings remained when analyzing behaviors at the individual level. Negative urgency did not moderate the relationship between brooding and behavioral dysregulation, and analyses of each individual dysregulated behavior revealed the same findings. Nonetheless, the present study provides clear supplementary support to the existing literature on the role of negative urgency across a variety of psychopathology symptoms in treatment‐seeking young people. This study also raises uncertainty about the role of brooding in dysregulated behaviors, and more broadly, in the ECM. While there was no evidence of brooding being implicated in behavioral dysregulation when accounting for negative urgency, further research on these interrelationships is required to clarify and understand their roles, and effectively tailor suitable treatment approaches.

## CONFLICTS OF INTEREST

The authors declare no conflicts of interest.

### PEER REVIEW

The peer review history for this article is available at https://publons.com/publon/10.1002/jclp.23366

## ETHICS STATEMENT

This study was performed in line with the principles of the Declaration of Helsinki. Approval was granted by the Deakin University Human Research Ethics Committee (DUHREC) on 06/12/2017 (No. 2017‐362). Informed consent was obtained from all individual participants included in the study. The authors affirm that human research participants provided informed consent for the publication of the current study.

## Supporting information

Supporting information.

## Data Availability

The data that support the findings of this study are available on request from the corresponding author. The data are not publicly available due to privacy or ethical restrictions.
