# Supplementary material for: Examining the role of brooding, distress, and negative urgency in dysregulated behaviors: A cross‐sectional study in treatment‐seeking young people
Source: J Clin Psychol. 2022 May 4;78(12):2538–63. doi: 10.1002/jclp.23366 (PMC9790647; doi:10.1002/jclp.23366)
Supplement: Supplementary file 1 — Supporting information. [file JCLP-78-2538-s001.docx]

**Appendix A**

Table A.1

*Model fit indices of individual latent variables*

|  | *n* | χ^2^ | *df* | RMSEA | CFI |
| --- | --- | --- | --- | --- | --- |
| Psychological Distress | 361 | 0.00*** | 0 | 0.00 | 1.00 |
| Brooding | 341 | 24.69*** | 5 | 0.11 | 0.95 |
| Reflection | 341 | 33.24*** | 5 | 0.13 | 0.92 |
| Negative Urgency | 337 | 5.06 | 2 | 0.07 | 0.99 |
| Behavioural Dysregulation | 358 | 5.93 | 3 | 0.05 | 0.98 |

*Note: *p*<.05; ***p*<.01; ****p*<.001;


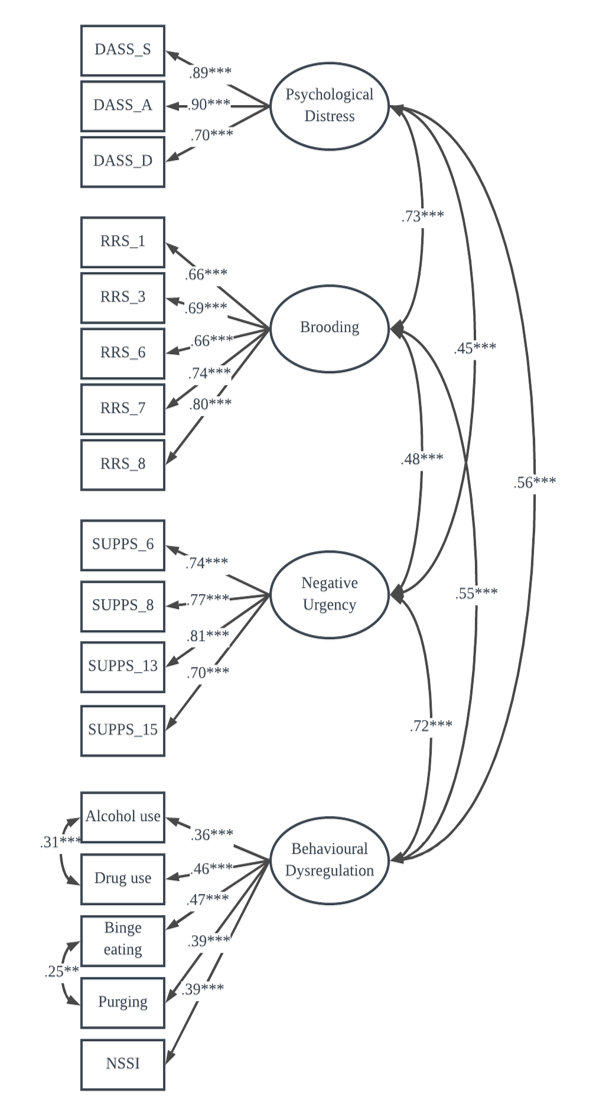
**Appendix B**

*Note:* PD: psychological distress; NU: negative urgency; NSSI:

non-suicidal self-injury; **p*<.05; ***p*<.01; ****p*<.001

*Figure A.1.* Measurement model of latent variables with standardised factor loadings and regression coefficients

**Appendix C**


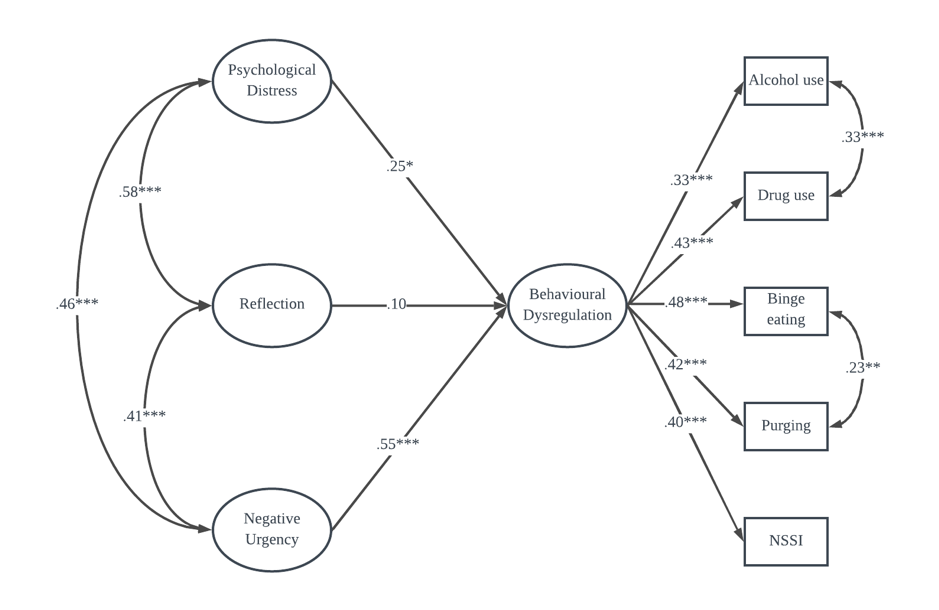


*Note:* NSSI: non-suicidal self-injury; **p*<.05; ***p*<.01; ****p*<.001

*Figure A.2*. Standardised factor loadings and regression coefficients for structural model demonstrating the relationship between psychological distress, reflection and negative urgency predicting behavioural dysregulation

**Appendix D**

Table A.2.

*Standardised beta weights with confidence intervals for latent variables predicting each dysregulated behaviour*

|  | Individual dysregulated behaviour | | | | |
| --- | --- | --- | --- | --- | --- |
|  | Alcohol use | Drug use | Binge eating | Purging | NSSI |
| Predictor | *β*  *95% CI*  [LL, UL] | *β*  *95% CI*  [LL, UL] | *β*  *95% CI*  [LL, UL] | *β*  *95% CI*  [LL, UL] | *β*  *95% CI*  [LL, UL] |
| PD | 0.05  [-0.09, 0.18] | -0.02  [-0.17, 0.13] | 0.14  [-0.04, 0.32] | 0.28*  [0.05, 0.51] | 0.22*  [0.03, 0.42] |
|  |  |  |  |  |  |
| Reflection | 0.02  [-0.13, 0.18] | 0.06  [-0.11, 0.23] | 0.12  [-0.07, 0.30] | 0.04  [-0.21, 0.29] | .04  [-0.18, 0.25] |
|  |  |  |  |  |  |
| NU | 0.28***  [0.13, 0.42] | 0.38***  [0.24, 0.53] | 0.28***  [0.13, 0.43] | 0.17  [-0.04, 0.38] | 0.26**  [0.09, 0.43] |
|  |  |  |  |  |  |

*Note:* PD: Psychological Distress; NU: Negative Urgency; NSSI: Non-Suicidal Self-Injury; **p*<.05; ***p*<.01; ****p*<.001.
